# Reimagining Remote Care: Advancing Telehealth to Support an Aging Population

**DOI:** 10.1093/geroni/igaf122.1898

**Published:** 2025-12-31

**Authors:** Walter Boot

## Abstract

As telehealth continues to evolve, it holds immense potential to support the health and well-being of older adults. However, barriers related to digital literacy, accessibility, and user preferences must be addressed to ensure equitable access and effectiveness. This symposium brings together experts to examine the challenges and opportunities of telehealth in diverse aging populations, with a focus on digital inclusion, usability, and the future of remote healthcare solutions. Dr. Sohyun Kim will explore the role of video chat interventions in Long-Term Services and Supports (LTSS) settings, highlighting their potential to enhance social engagement and family relationships for persons living with dementia while addressing technological and logistical barriers. Sungjae Hong, MS, MPH, will present an exploratory study on telehealth adoption among Korean older adult immigrants, revealing key factors influencing digital healthcare use and the role of healthcare providers in shaping adoption. Dr. Paul P. Freddolino will introduce a comprehensive cost-benefit model assessing the impact of telehealth awareness and digital literacy training for direct care workers, examining the economic, social, and functional value of these initiatives for multiple stakeholders. Dr. Renato Ferreira Leitao Azevedo will discuss findings from an online feasibility study investigating older adults’ preferences for different telehealth communication mediums, evaluating their effectiveness in enhancing comprehension, emotional engagement, and risk perception. By addressing critical issues in telehealth adoption and design, this symposium will provide valuable insights into optimizing remote care for aging populations.

